# Members’ experiences and perceptions of participating in an Australian Regional One Health Network

**DOI:** 10.1186/s42522-023-00093-2

**Published:** 2024-01-11

**Authors:** Kirrilly Thompson, Joanne Taylor, Peter D. Massey, David N. Durrheim

**Affiliations:** 1 Hunter New England Local Health District, Wallsend, 2287 Australia; 2 School of Public Health, Medicine and Well-being, University of Newcastle, Callaghan, 2308 Australia; 3 Flinders Health and Medical Research Institute, Flinders University, Bedford Park, 5042 Australia; 4 School of Health, University of New England, Armidale, 2350 Australia

**Keywords:** Collaboration, Membership survey, One health, Network, Survey tool, Policy, Wordcloud, New South wales, Australia, Welfare, Health

## Abstract

**Background:**

The One Health framework is intended to optimise the interdependent health of humans, animals and ecosystems. It relies on effective collaborations across disciplines, sectors and communities. One Health networks have become increasingly important platforms for encouraging, creating and supporting collaborations. Their success is usually judged by evaluations of their outputs. However, there is also a need to understand member experiences and perceptions of the networks in which they participate. To that end, we undertook the first membership survey of a One Health network that was established in Australia in 2005.

**Methods:**

An online membership survey was created, comprising closed and open-ended questions.

**Results:**

Around one third of the Regional One Health Partnership (‘the Network’) participated in the study (33 members). Participants contributed a combined total of 170 years of experience in the Network and 414 years of combined experience working in/on One Health. The Network has provided excellent opportunities for cross sectoral collaboration that would otherwise not have been possible. Findings also highlighted the intangible benefits of membership such as the creation of a collaborative support group for emerging and established One Health practitioners.

**Conclusions:**

The Network plays an important role in One Health collaborations in New South Wales and further afield. Commensurate with the literature on One Health collaborations globally, we identified a need for greater diversity amongst members, especially from First Nations people, local communities, non-government organisations and wildlife/environment experts, as well as concerted attempts to identify policy implications. Our membership survey tool could be adapted for future One Health Network membership surveys in Australia and internationally.

**Supplementary Information:**

The online version contains supplementary material available at 10.1186/s42522-023-00093-2.

## Introduction

Climate change is one of the most serious threats of our time, alongside (and related to) emerging infectious diseases, antimicrobial resistance, and food and nutrition insecurity [1]. All of these threats are One Health issues, representing interlinked human, animal and environmental origins, interactions and implications. The One Health High Level Expert Panel recently clarified the definition of One Health as ‘an integrated, unifying approach that aims to sustainably balance and optimize the health of people, animals, and ecosystems. It recognizes the health of humans, domestic and wild animals, plants, and the wider environment (including ecosystems) are closely linked and interdependent’ [2]. The importance of One Health has been underlined by the COVID-19 pandemic [1, 3], with probable zoonotic origins likely related to human land use [3]. This importance is further accentuated by copious pathogen spillover events, with an analysis of 335 zoonotic emerging infectious disease events between 1940 and 2004 finding that 71.8% were caused by pathogens with a wildlife origin [4]. The consequences of the COVID-19 pandemic, and other spillover events, extend beyond loss of human life to significant psycho-social and economic detriment [5]. Effectively addressing and mitigating One Health problems requires – at the least, effective multidisciplinary and multisectoral collaborations involving experts and stakeholders in the health of humans, animals and the environment [6]. More cohesive transdisciplinary collaborations can be achieved by additional input from other scientific paradigms such as the humanities, and/or from non-scientists and other partners [7], as illustrated by one collaboration between medical anthropology, epidemiology and preventive medicine [8].

Around the same time that the ‘One Health’ concept was gaining momentum [9], One Health networks started to proliferate, as evidenced by a systematic review of 100 unique One Health networks in Africa, Asia and Europe [10]. Recently, Mwatondo and colleagues documented 184 One Health Networks [6]. The most well-known is the tripartite, involving the World Health Organization, Food and Agriculture Organization of the United Nations and World Organisation for Animal Health in 2010 [11], and the 2022 addition of the United Nations Environment Programme to form the Quadripartite.

In 2005, a regional One Health network was formed in the Hunter New England region of New South Wales (NSW), Australia. It was named the Regional One Health Partnership, referred to in this paper as the ‘Network’. The Network did not form for the purposes of addressing a particular project or specific One Health issue, as is the case with many networks [6]. Rather, it arose from shared interests in zoonotic disease prevention, and animal health concerns relevant to regional northern NSW, initially with a focus on Q Fever, *Leptospirosis*, and *Cryptosporidium* [12]. The Network was initiated by Authors 3 and 4 along with a senior epidemiologist from the Hunter New England Public Health Unit in collaboration with several veterinarians. Initially, the Network was focused on the Hunter New England region. However, it quickly expanded with the broad geography covered by members, especially those in adjoining local health districts. Similar networks arose in southeast Queensland and Victoria, from around 2017.

The Hunter New England Area of northern NSW is a diverse area covering 130,000 km^2^. Along the coastal margins, population density is reasonably high with concentrated urban development. Large national parks and untouched woodland covers the Great Dividing Range, a rugged area parallel to the coast. In the easterly formations of the Great Dividing Range is the Hunter Valley, home of Australia’s oldest wine growing region and Thoroughbred horse breeding capital (the second largest in the world), combined with a long history of industrial and mining activity that all likely impacted on biodiversity. To the immediate west of this range is fertile agricultural land with many small towns. The remote western areas comprise arid country suited to low-intensity livestock-farming which is also home to wild native and feral animals. The geography, demography, agriculture and recreational activities of northern NSW provide many opportunities for human-animal interactions with the possibility for shared diseases. Outbreaks of avian and equine influenza, Q fever, babesiosis, salmonellosis, cryptosporidiosis, Hendra virus and various arboviral diseases have all occurred since 2005. Avian influenza outbreaks in poultry facilities are an ongoing concern as the Hunter New England region also includes a large poultry industry. There was a mouse plague in 2021, coinciding with the COVID-19 pandemic and detections of Japanese encephalitis in 2022.

The aims of the Network - as outlined in the Terms of Reference, are:

1. To provide a network for facilitating communications to professionals working in the general area of One Health.
2. To encourage and facilitate One Health projects, special interest (working) groups and activities in the One Health space.
3. To identify reference people to assist with One Health matters.
4. To share information, knowledge, experience and enthusiasm.
5. To shape the regional research agenda.
6. To contribute to the reduction of risk and improvement of the health of people, animals and the environment in the regional area.

From the outset of the Network, any interested individual without political or pecuniary interests was welcome to participate. Initially, it involved partnership with the Department of Primary Industries. As the Network developed, additional members were invited to join or requested membership. In 2015, Network members represented NSW Health - Public Health, the Department of Primary Industries, Local Land Services, Wildlife Health Australia, and the Australian Veterinary Association. By 2018, the network involve clinicians, veterinarians, academics, epidemiologists, public health practitioners, environmental scientists, wildlife health specialists, pathologists and others [13]. In 2019, 13 agencies were participating in the Network.

The Network has continued to expand for collaborative purposes, and some members still participate in the Network after moving to new locations and organisations. This wide collaboration is important given that there is overlap in One Health issues in Northern NSW and South-eastern and South-western Queensland. Some of the initial members have retired but still maintain a connection to the Network.

Currently, there are around 90 members. Meetings are held quarterly via teleconference. Each meeting includes facilitated discussion following updates from human, animal and ecological perspectives. Attempts are made to have a presentation and/or update from each of the three main facets of One Health – human, animal and ecological health. Meetings are typically attended by 30–40 members. Three multi day face-to-face workshops have also been held; two in Hunter New England and one in Queensland. In between teleconferences, informal email communication is encouraged via the group email list. This includes sharing articles, notifications of conferences, requests for collaboration, and specific disease risks or health emergency warnings.

Since its inception, outputs resulting from Network collaborations have included research projects/publications, reviews of various policy documents/factsheets, joint exercises and outbreak responses (e.g. avian influenza), combined public messaging, shared debriefs/reports, shared data (e.g. salmonella isolations), pooled expertise, student placements and prompt incident alerts. Indicators for the success of the Network include these kinds of outputs and activities, in combination with its continuance over 18 years (which may be attributable to consistent leadership). However, there is also a need to determine members’ own experiences and perceptions of Network benefits.

## Aims

The primary aim of this study was to determine members’ experiences and perceptions of their participation in the Regional One Health Network. We sought to understand experiences in terms of commitment (years of Network involvement), how people found out about the Network and their motivations for joining. We also sought to understand participants’ perceptions of the benefits of participation, the extent to which the Network had provided them with exceptional opportunities and collaboration, and the usefulness of the Network – especially regarding being informed, forming new collaborations, strengthening existing collaborations, and overall usefuless. Whilst contributions to policy change were not formalised in the written Terms of Reference reproduced above, they were included in the survey tool to capture this important aspect of One Health partnerships. A secondary aim was to pilot a One Health Network membership survey tool for understanding Network membership, identifying areas for improvement, and assessing changes in experience and perception over time.

## Materials and methods

An online membership survey was developed, reviewed and piloted by several members of the network (see supplementary material). It covered:

1. Experience working in One Health.
2. Experience of the Regional One Health Network.
3. Current One Health projects.
4. Perceived benefits, impact and outcomes of participating in the Regional One Health Network.
5. Thoughts on the future of the Regional One Health Network.
6. Demographic information including professional qualifications and type of employment/organisation.

In this article, we report on all of the above items except for Item 5, which allowed participants to comment on what they would like to achieve, what they wanted the Network to achieve, how the Network could assist them, how the Network could be improved, and their preferred forms of communication. The findings from these questions are not presented here, as they were considered too sensitive/identifying and/or beyond scope of this report. However, they have been discussed internally between members at meetings, with the development of an action plan.

At the start of the survey, participants were reminded of the US Center for Disease Control’s definition of One Health as ‘…an approach that recognizes that the health of people is closely connected to the health of animals and our shared environment’ [14].

All questions were optional and branching logic was used where relevant. There was a mixture of question types including closed-ended, multiple-choice, Likert scale and free-text responses. After confirming consent to participate, no questions were compulsory and participants were able to continue after skipping any question, hence the inclusion of response rate alongside findings for individual questions throughout this article.

Data were collected and managed using REDCap electronic data capture tools hosted at Hunter New England Local Health District.^1^ The survey link took potential participants to an information statement and informed consent page. The study protocol received approval from the University of Newcastle Human Research Ethics Committee (H-2022-0203). Consent was implied by the submission of the anonymous online survey. The study was undertaken according to the Australian National Statement on Ethical Conduct in Human Research [15].

## Recruitment

An invitation to participate was emailed to 89 Network members on 5 May 2022. Reminders were made in invitations to Network meetings in May, June, September and December 2022, as well as during those meetings. The last survey was completed on 4 November 2022.

## Analysis

Quantitative data from closed-ended questions were analysed using descriptive statistics. As the aims of the present paper are largely deductive - with survey questions clearly aligned to address the research questions - qualitative data from open-ended questions were not subject to a process of systematic inductive analysis that would be characterised by coding and the abstraction of themes [16]. Rather, categorisation was sufficient to summarise free-text response. Open-ended responses were read by Author 1, assigned to a categorical ‘code’, and presented to the other authors for discussion and agreement. Illustrative quotations are presented for the reporting of open-ended questions where they provide additional insight and to illustrate the breadth of responses.

Where the coding of free-text responses would have resulted in an excessive list of unique codes, and where aggregation of those codes would have undermined the intention to provide breadth and depth, responses are represented visually in word clouds. Larger font sizes indicate higher frequency amongst responses, although frequency is also reported numerically alongside words. Word clouds were generated using the online word cloud generator [17]. Prior to uploading text to the generator, textual data were ‘cleaned’ in two ways. First, all words considered irrelevant to the word cloud product were removed, such as: in, of, during, mostly. Second, separate words that needed to be treated as a ‘whole’ (e.g. ‘emerging infectious disease’) were edited with the insertion of a tilde (~) which functioned as a non-breaking space in the cloud. Original wording was maintained as much as possible to increase validity and preserve diversity, hence several specialisations in a similar theme may be visible in a world cloud (for example, ‘surveillance’, ‘surveillance system’ and ‘surveillance program’ in Fig. 3). The alphabetical listing of words in clouds makes it possible to quickly identify similar items. Whilst it is not uncommon to present data in ‘word’ or ‘tag’ clouds [18], they should be interpreted as a visual indication of prevalence, especially as they treat synonyms separately [19].

## Results

### Participants

The consent question was completed by 33 Network members, including the authors. All questions in the survey tool were completed by 26 participants. Seven participants left at least one question unanswered.

The main demographics for participants are presented in Table 1.

**Table 1:** Main demographics of participants in the Regional One Health Partnership (the Network)

Demographic	Value	Count, %
Gender (n = 26)	Female	18, 69%
	Male	7, 27%
	Non-binary	0
	Prefer not to say	1, 4%
Qualifications relevant to One Health (n = 26)	PhD	15, 58
	Undergraduate/bachelor’s degree	15, 58%
	Master’s degree	7, 27%
	Diploma	3, 12%
	Certificate	3, 12%
	Honours degree	1, 4%
	Other (medical and veterinary specialisations and higher degrees)	3, 12%
Organisation classification (n = 25)	State/Territory government	12, 48%
	Higher education	9, 36%
	Federal government	3, 12%
	Not for profit/advocacy group	2, 8%
	Other (clinicians in a public hospital and a veterinary practice)	2, 8%
	Local government or council	1, 4%
	Private industry	0
	Personal business/consultant	0^2^, 0%
Role in organisation (n = 26)	Research	13, 50%
	Education and/or training	13, 50%
	Clinical	11, 42%
	Role fostering collaborations	9, 35%
	Program development	7, 27%
	Prevention	7, 27%
	Policy	5, 19%
	Other (Emergency management, higher degree research training, diagnostic/consultation, surveillance and response)	4, 16%
Sector (n = 26)	Human health	17, 65%
	Animal health	17, 65%
	Environmental health	9, 35%
	Wildlife health	8, 31%
	Cross cutting across sectors	8, 31%
	Ecology	6, 23%
	Other (zoonotic disease)	1, 4%
Geographic region or area of relevance (n = 25)	New South Wales	7, 28%
	Hunter New England	6, 24%
	Australia	5, 20%
	Northern NSW	4, 16%
	International	3, 12%
	Victoria	2, 8%
	Coastal areas, Eastern Australia, Northern Tablelands, Narrabri local government area (LGA), the Pacific, Papua New Guinea, Queensland, Riverina, South-Eastern NSW, urban NSW, Walgett LGA, wildlife free-range areas.	For each value: 1, 4%

### Area/s of expertise

**Fig. 1:**
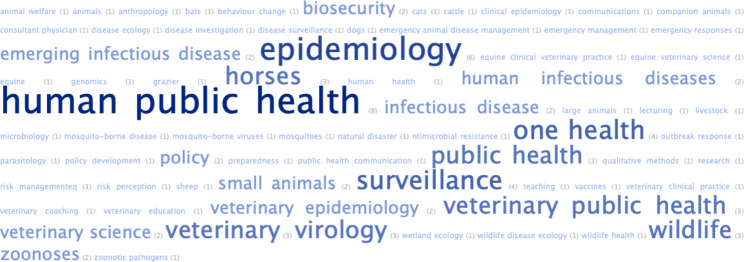
Word cloud representation of participants’ area/s of expertise (n = 26)

Participants were asked to describe their area of expertise in free-text. Examples provided with the question were ‘large animals, small animals, horses, dogs and cats, wildlife, human public health, surveillance, epidemiology, policy, etc.’ There were 59 unique responses amongst 243 words. This breadth illustrates a diversity of expertise amongst the 26 members who answered this question (Fig. 1).

### Position in organisation

**Fig. 2:**
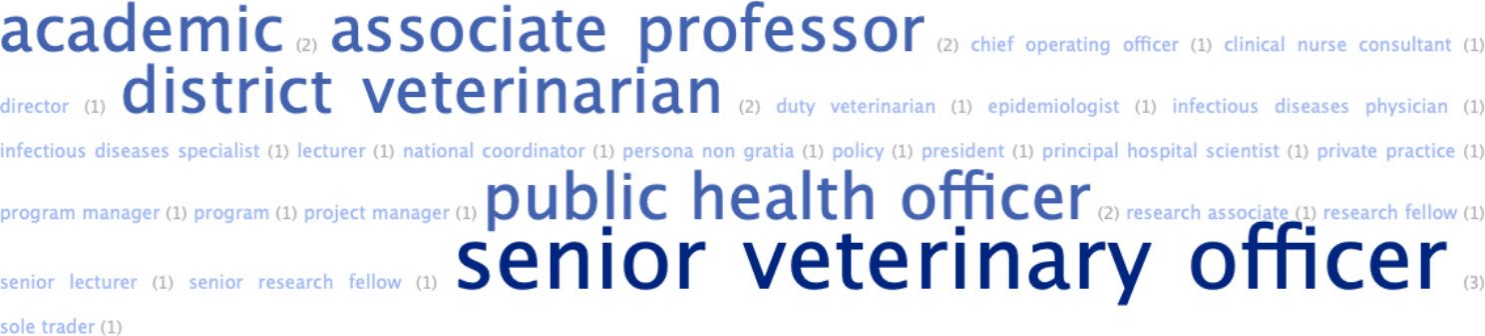
Word cloud representation of participants’ position in their organisation (n = 25)

Participants were given a free-text opportunity to state their position in their organisation. There were 22 unique responses amongst 82 words (see Fig. 2).

### Main One Health projects/programs/topics

**Fig. 3:**
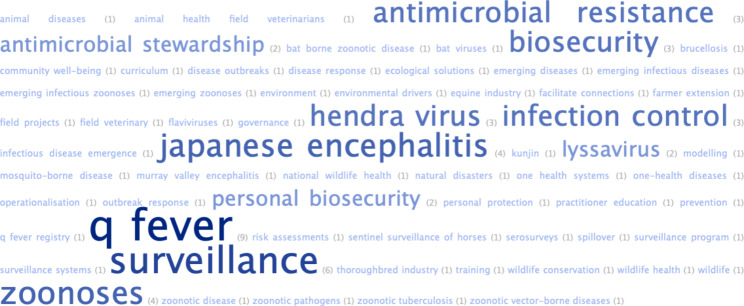
Word cloud representation of main One Health projects/programs or topics on which participants were working (n = 29)

Participants were asked to list the main one health projects/programs/topics on which they were currently working. The responses from 29 participants are illustrated in Fig. 3.

Participants were asked to select one or more areas of concern related to their current One Health work, from a pre-determined list. From responses provided by 31 participants, their work related to: human health (25, 81%), One Health surveillance (23, 74%), wild animal health (20, 65%), livestock health excluding horses (19, 61%), One Health systems (18, 58%), horses (18, 58%), domestic animal/pet health (17, 55%), acute disease (16, 52%), endemic disease (15, 48%) and the environment (14, 45%). One participant (3%) selected ‘other’ and explained that their project/program was related to “exotic disease, emerging disease”.

Nineteen participants indicated in free-text responses that they were actively seeking collaboration on at least one project or program of work related to One Health. They were prompted to include in their response the kind of assistance required such as disease, issue, methodology, region, field of work, or species. The responses conveyed across 422 words are too identifying or detailed to effectively quantify with a content analysis or illustrate in a word cloud. However, they do reflect potential for at least nineteen further Network collaborations. The following quotation demonstrates the willingness of members to collaborate and the opportunity provided by the Network.

Always seeking collaborators, but no specific projects to list here. Will raise with group as they arise (P22).

### Years of experience working in one health

The range of years working on One Health topics/issues across participants was 0 to 37 years with a mean of 13.4 and a median of 10.0 years. Together, the 31 participants responding to this question contributed a total of 414 years of experience working in One Health to the Network.

### Experiences of the regional one health network

Years involved in the Regional One Health Network and One Health generally.

Thirty people responded to a question asking how many years they had been involved in the Network. Responses ranged from 0 to 20, the latter of which would have been an estimate as the survey tool did not specify the start date of the Network as 2005. There was a mean of 5.7 years of involvement with a median of 4.0. Together, participants had a total of 170 years of experience in the Network.

### How did you find out about the regional one health network?

A free-text question asking participants to describe how they found out about the Network was addressed by 30 people. Almost half found out via a colleague, word of mouth or invitation/recommendation from a specific person (17, 50%). Five (15%) were original members or Network founders. Three had been invited to present at a meeting (9%), were referred by another group (9%) and/or the Department of Primary Industries (6%). Two could not remember (6%). One learnt about the Network from a Public Health Bulletin (3%) or found out via email (3%).

### Reason(s) for joining the network

Free-text responses from 30 participants about their reason for joining the Network totalled 524 words, including multiple reasons, which were allocated to nine different categories. One third joined to build relationships, networks, connections, partnerships and collaborations (19, 33%). For example, one participant wrote:

To build relationships to work effectively in responses and build consistent messaging across human and veterinary health (P2).

Over a fifth joined to maintain awareness of current/key issues (13, 23%). As one participant wrote,

There is a need for those of us that treat zoonotic infections in humans to have a greater understanding of those diseases that can only come with collaboration with our veterinary and scientific colleagues (P20).

Almost one fifth joined because of their desire to form collaborations (10, 18%) or interest in One Health (10, 18%). Regarding the latter, one participant wrote,

Interest in One Health as an approach to healthcare of humans and animals, as well as recognising the vital role of environmental sustainability and preservation (P28).

Six people were interested in the regional focus of the Network (11%), whilst two were looking for a like-minded group (4%). One person was interested in information sharing and learning (2%), another for work opportunities (2%) and another due to their respect for others in the Network (2%). In addition to these nine reasons for joining the Network, there were two unique mentions of specific diseases as a reason for joining: cryptosporidium (2%) and Japanese Encephalitis (2%).

### Perceptions of the regional one health network

#### Benefits of the regional one health network

Participants were asked to describe the major One Health-related benefits they had gained from the Network in the past five years, with instructions to list up to three benefits. Responses were considered too detailed to be pared back to keywords to produce a word cloud (495 words from 28 participants). However, they can be summarised as referencing the anticipated benefits of One Health collaborations including collaboration, networking, knowledge-sharing, dissemination, multidisciplinary contextualisation and systems-based understandings, as well as being kept informed. For example, the following comment illustrates the valuable social and professional support provided to a PhD candidate participating in the Network:

Interest in my PhD research in OH including broadening the reach of my research findings. The group was actively interested in what I and other PhD students were doing which I found very encouraging, particularly as many of us were from a background in veterinary science (P8).

That sentiment was echoed in comments made at the end of the survey in response to a standard ‘any other comments’ question, such as:

Wonderful to see this initiative flourish for this period of time - must be meeting a need (P1).

Find it a wonderful example of how a group of enthusiastic and dedicated people can raise the profile of and make a change in the One Health space. A great template for other groups to develop in other regions (P2).

A respectful group I personally feel a valued member of the group (P15).

#### Provision of exceptional One Health opportunities and collaboration

Participants were asked if the Network had provided them or their institution with ‘exceptional’ One Health opportunities that would have otherwise not been possible. Three quarters of 28 responses indicated ‘yes’ (21, 75%). Nineteen people described the opportunity to which they were referring, with eleven directly referencing ‘collaboration’.

When specifically asked if being a part of the Network had enabled them to develop any collaborations since they joined, more than half indicated ‘yes’ (17, 61%). The following insight was provided in responses to a branched free-text question asking participants to detail how those One Health Network collaborations had been initiated:

Direct contact with persons, researchers, departments, agencies. The benefit in knowing who to contact (P12).

[For] collaboration 1: I volunteered to collaborate on one publication on a topic I was interested and had some knowledge to contribute to. [For] collaboration 2 (2 studies): I was invited to collaborate on a project for which I had expertise on the topic and methodological approach (P18).

First-name familiarity with other people who I feel able to approach to discuss project ideas or invite for collaboration (P31).

One collaboration after invitation for the expert to present at the One Health meeting (IPC expert) (P33).

#### Usefulness of the One Health Network

Using a four-point Likert scale, participants were asked to indicate (a) their level of agreeance with five statements about the benefits of the One Health Network, and (b) the overall usefulness of the network.

Most participants agreed that the network had greatly benefitted being informed, forming new collaborations, and strengthening existing collaborations. Almost half (13, 48%) noted an impact on policy. All participants perceived the overall usefulness of the Network positively (see Table 2).

**Table 2:** Participant agreement with statements about the benefits of the One Health Network (n = 27)

Statement/Question	Value	Count, %
The Regional One Health Network has assisted me/my institution with being INFORMED of relevant One Health issues	Greatly	23, 85%
	Somewhat	4, 15%
	Not at all	0
	Unsure	0
The Regional One Health Network has assisted me/my institution with FORMING new One Health collaborations for research, surveillance or outbreak response	Greatly	13, 48%
	Somewhat	11, 41%
	Not at all	2, 7%
	Unsure	1, 4%
The Regional One Health Network has assisted me/my institution with STRENGTHENING existing One Health collaborations for research, surveillance or outbreak response	Greatly	15, 56%
	Somewhat	8, 30%
	Not at all	2, 7%
	Unsure	2, 7%
The Regional One Health Network has contributed to POLICY CHANGE in my sector	Greatly	3, 11%
	Somewhat	10, 37%
	Not at all	4, 15%
	Unsure	10, 37%
Overall, how would you describe the usefulness of the Regional One Health Network to you?	Very useful	21, 78%
	Fairly useful	5, 19%
	A little bit useful	1, 4%
	Not at all useful	0

## Discussion

The participation of 33 Network members in our study reflects typical attendance at quarterly teleconferences and represents over one third of people on the Network email communications list. To gain this level of representation, the survey remained open for a period of six months. Reminders to participate were made by email and reminders were made verbally during meetings and included with notifications of – and reminder for, upcoming meetings.

There is a recognised tendency for the environmental pillar of One Health to be overshadowed by human and animal health [1, 20, 21]. In our Network, animal and human health roles and sectors were generally equally distributed for at least half of all participants. Ecology and wildlife were relatively less well represented amongst the Network, although over half said that the One Health projects/programs/topics on which they were currently working concerned wild animal health (20, 65%).

There have been calls for more involvement of the social sciences in One Health partnerships [6, 21], including geographers and anthropologists [8, 22]. The inclusion of an anthropologist in our Network (Fig. 1) demonstrates progress in this regard, especially considering that anthropology is an inherently holistic and anti-reductionist discipline [23]. However, more members with backgrounds in the social sciences could be involved – not only to avoid tokenism, but to maximise the advantages of collaborations between multiple disciplines. There is also an important role for the humanities to be involved in One Health, especially to address the political, philosophical, moral and ethical dimensions of One Health that are receiving increasing attention [24, 25].

Other voices that are all too commonly absent from One Health networks include First Nations representatives, community stakeholders and the private sector [6]. Whilst our survey did not ask participants if they were Aboriginal or Torres Strait Islander, the Network could more deliberately include First Nations voices, as well as involve people representing communities and non-government organisations. There appear to be existing social networks and pathways for increasing participation from these under-represented groups, based on our finding that half of participants found out about the Network from others, via word of mouth, recommendation or were invited by existing members.

Findings suggest that the Network has facilitated One Health collaborations, as evidenced by three-quarters believing the Network had provided them or their institution with exceptional One Health opportunities that would otherwise not have been possible. Indeed, all participants believed that their membership in the Network was valuable for keeping them informed of relevant One Health issues, and most reported value in forming new and strengthening existing collaborations (Table 2). The benefits of the Network to policy change were least reported by participants.

However, not all projects or programs of One Health work have policy implications. In fact, policy was not mentioned in responses to questions asking about: reasons for joining the Network, major One Health benefits gained from the Network or exceptional One Health opportunities. This was despite two participants mentioning policy when describing their area of expertise. Still, the relatively low impact of Network participation on policy (relative to other predetermined benefits), together with the absence of policy in free-text responses does not necessarily mean that policy is irrelevant to members and not impacted by Network participation. Therefore, future surveys should seek to determine participants’ desire and ability to influence policy. For example, an option to select ‘policy’ could be added to the question asking participants about their role in their organisation. An open-ended question could also be included asking participants selecting ‘policy’ to elaborate, as was asked in relation to collaborations. Finally, it may be more appropriate for policy change to be judged by more senior members of each organisation represented in the Network. Notwithstanding, the inclusion of policy in the Network Terms of Reference will be discussed at future meetings.

A secondary aim of this study was to develop and pilot a tool for membership surveys with similar One Health networks. The survey design included a mixture of closed and open-ended questions. Closed-ended questions allowed the collection of information that was expected in relation to the research aims, but the addition of open-ended questions provided an opportunity to collect unanticipated information and contextualise responses to closed-ended questins. There was some overlap whereby closed and open-ended questions were effectively asking the same question. For example, whilst one open-ended question asked participants to describe the benefits they had received from their participation in the Network, four anticipated benefits were also provided as statements to which participants could show their agreement via a Likert scale (Table 2). The fact that similar responses were provided to the open-ended question as the closed-ended Likert scales provided triangulation to support the validity of our finding that the Network was beneficial for keeping members informed (i.e. knowledge-sharing, dissemination, being up to date), and forming new and strengthening existing One Health collaborations (i.e. collaboration, networking).

Some open-ended questions provided responses with a breadth of detail that was challenging to summarise without oversimplification. In a One Health context of multi-, inter- and trans- disciplines and sectors, questions about seemingly straightforward things like expertise and position in an organisation can have complex answers. Even responses from a small group of participants can be incomparable, as occurred with responses to the question asking participants to describe their main One Health projects, which included: type of disease (e.g. zoonotic, human or animal), type of animal (e.g. bat, horse, Thoroughbred horse), type of project (e.g. surveillance or extension), type of approach (e.g. prevention or response) and type of outcome (e.g. curriculum or partnerships). Word clouds proved useful for capturing and conveying the breadth and saturation of such responses. However, future iterations of the survey may seek to disaggregate these different types of responses into separate questions focused on type of disease, type of approach, etc. and triangulate each with a clarifying statement about the specific *point d’entrée* (e.g. animal-centric, human-centric, environment-centric or genuine transdisciplinary approach).

Some of the categorisation of free-text presented in this article could also be integrated into multiple-choice responses in future membership surveys of this and other One Health networks, which could make the survey more efficient for participants (e.g. how people found out about the Network and/or their reason for joining). An ‘other’ option would still be recommended with this and all multiple-choice questions, to maintain the ability to collect unanticipated responses. Nonetheless, some of the free-text responses provided insight into the intangible and unquantifiable value of the Network, such as the creation of a welcoming environment which is especially for people who feel like they are only at the start of their One Health journey, such as PhD candidates. Indeed, the creation of a safe and welcoming space for individual confidence to flourish and interpersonal relationships to develop is essential for collaboration - arguably even more so than professional leadership skills [26]. Based on that kind of valuable insight from this study, we would encourage the inclusion of an open-ended ‘benefits’ question in future membership surveys. Whilst the survey design reported in this study could be modified for one-on-one interviews, the relative depth of exploration that they provide may be more suited to reporting findings from smaller One Health networks. Nonetheless, options for participants to choose interview or survey format may increase participation rates. Finally, given that our survey tool included the United States CDC definition of One Health, we recommend that future studies include the 2022 OHHLEP definition of One Health [2].

Our findings should be considered in the context of limitations associated with self-report, self-administered survey designs [27]. Additionally, as the research team included two respected founding members of the group, there was a possibility of a social desirability bias towards positive appraisals of the Network. However, when promoting the survey link to Network members during meetings and in written communication, we made it clear that we were looking for honest feedback for the purposes of improving the Network. In our survey, the four authors were also participants. The pros and cons of self-participation were discussed at length amongst the team. Given the unique backgrounds and experiences of the authors, their experiences and opinions were considered important to the diversity of responses. The range of responses provided in Table 2 are not suggestive of a strong social desirability or acquiescence bias, especially with lower levels of agreement around the impact of Network participation on policy. Notwithstanding these limitations, findings confirm that a self-report survey was appropriate relative to our aims of determining members’ experiences and perceptions.

## Conclusion

In this paper, we conducted a membership survey of a regional One Health network in New South Wales. Founded in 2005, the Network continues to provide One Health benefits, especially collaborations between disciplines and sectors that are essential for impactful One Health outcomes and which would otherwise be unlikely. To continue to benefit the health of humans, animals and the environment in New South Wales and beyond, we recommend an explicit strategy of increasing the diversity of members to include representation from First Nations people, wildlife/ecology, the community and not-for profit sectors. We also suggest that more consideration be given to the potential impact of the Network on policy. Our study has provided a piloted membership survey tool and guidance that could be adapted by other One Health networks in Australia and internationally to determine member experiences and perceptions.

### Electronic supplementary material

Below is the link to the electronic supplementary material.

**Supplementary Material 1**: Regional One Health Network Survey Tool 2022

## Data Availability

The datasets used and/or analysed during the current study are available from the corresponding author on reasonable request.

## Footnotes

1 REDCap (Research Electronic Data Capture) is a secure, web-based application designed to support data capture for research studies, providing (1) an intuitive interface for validated data entry; (2) audit trails for tracking data manipulation and export procedures; (3) automated export procedures for seamless data downloads to common statistical packages; and (4) procedures for importing data from external sources.

2 However, one participant described their position as ‘sole trader’ in their response to another question (see Fig. 2).

## List of abbreviations

NSW: New South Wales
REDCap: Research Electronic Data Capture

## References

[1] Lancet The (2023). One health: a call for ecological equity. The Lancet.

[2] Expert P, One Health High-Level (2022). One health: a new definition for a sustainable and healthy future. PLoS Pathog.

[3] Lefrançois T et al. *After 2 years of the COVID-19 pandemic, translating one health into action is urgent*. The Lancet. 10.1016/S0140-6736(22)01840-2 PMC9595398 36302392

[4] Jones KE (2008). Global trends in emerging infectious Diseases. Nature.

[5] McBride O (2021). Monitoring the psychological, social, and economic impact of the COVID-19 pandemic in the population: Context, design and conduct of the longitudinal COVID‐19 psychological research consortium (C19PRC) study. Int J Methods Psychiatr Res.

[6] Mwatondo A, et al. A global analysis of one Health networks and the proliferation of one health collaborations. The Lancet; 2023. 10.1016/S0140-6736(22)01596-3 36682370

[7] Errecaborde KM (2019). Evaluating one health: the role of team science in multisectoral collaboration. Revue Scientifique et Technique de l OIE.

[8] Ortega C, Ortega J, Simón MC (2022). Anthropology and One Health: a Transdisciplinary Approach to understanding Diseases Emergence. Open Access Library Journal.

[9] Vallat B. *One World, One Health* Editorials from the Director-General. Publisher: World Organisation for Animal Health (oie), 2009. 2: p. 1–2.

[10] Khan MS (2018). The growth and strategic functioning of one health networks: a systematic analysis. Lancet Planet Health.

[11] World Health Organization. Food and Agriculture Organization of the United Nations, and World Organisation for Animal Health. Taking a multisectoral one health approach: a tripartite guide to addressing zoonotic Diseases in countries. Food & Agriculture Org; 2019.

[12] Ng J (2008). Evidence supporting zoonotic transmission of Cryptosporidium in rural New South Wales. Exp Parasitol.

[13] Eastwood K (2018). Unravelling zoonotic Diseases in Australia. Australian J Gen Pract.

[14] Centers for Disease Control and Prevention. One Health Basics. 2022 [cited 2023 02 January]; Available from: https://www.cdc.gov/onehealth/basics/index.html.

[15] National Health and Medical Research Council (2007). National statement on ethical conduct in human research.

[16] Green J (2007). Generating best evidence from qualitative research: the role of data analysis. Aust N Z J Public Health.

[17] Steinbock D. *TagCrowd*. [cited 2023 02 January]; Available from: https://tagcrowd.com.

[18] Paterson BJ, Durrheim DN (2013). The remarkable adaptability of syndromic surveillance to meet public health needs. J Epidemiol Global Health.

[19] Atenstaedt R (2017). Word Cloud analysis of the BJGP: 5 years on. Br J Gen Pract.

[20] Essack SY (2018). Environment: the neglected component of the one health triad. Lancet Planet Health.

[21] Humboldt-Dachroeden S, Rubin O, Sylvester Frid-Nielsen S (2020). The state of one health research across disciplines and sectors – a bibliometric analysis. One Health.

[22] Giraudoux P et al. *One Health or “One Health washing”? An alternative to overcome now more than ever*. Vol. 2022. 2022: CABI International. 4.

[23] Harkin ME (2010). Uncommon ground: Holism and the future of Anthropology. Reviews in Anthropology.

[24] Degeling C (2015). Implementing a one health approach to emerging Infectious Disease: reflections on the socio-political, ethical and legal dimensions. BMC Public Health.

[25] Degeling C, Kerridge I (2013). Hendra in the news: public policy meets public morality in times of zoonotic uncertainty. Soc Sci Med.

[26] Stephen C, Stemshorn B (2016). Leadership, governance and partnerships are essential one health competencies. One Health.

[27] Ponto J (2015). Understanding and evaluating survey research. J Adv Practitioner Oncol.

