# Supplementary material for: Members’ experiences and perceptions of participating in an Australian Regional One Health Network
Source: One Health Outlook. 2024 Jan 11;6:2. doi: 10.1186/s42522-023-00093-2 (PMC10782705; doi:10.1186/s42522-023-00093-2)
Supplement: Supplementary file 1 — Supplementary Material 1: Regional One Health Network Survey Tool 2022 [file 42522_2023_93_MOESM1_ESM.pdf]

R1HN Survey 2022

PARTICIPANT INFORMATION STATEMENT

for the project titled 'A survey-based evaluation of the Regional One Health Network'

Introduction
You are being invited to participate in this survey because you are a current member of the Regional One Health Network.

The purpose of this survey is to learn more about current members of the Regional One Health Network, evaluate the benefits of the Regional One Health Network and identify further opportunities and possibilities. In particular, we want to:

- **Describe the people participating in the Regional One Health Network**
- **Determine the motivations/drivers for participation**
- **Document the value of the Regional One Health Network**
- **Document impact and outcomes related to participation in the Regional One Health Network**
- **Identify areas for further development of the Regional One Health Network**
- **Support further collaborative work by and between Regional One Health Network members.**

In this survey, we use the CDC definition of One Health as '...an approach that recognizes that the health of people is closely connected to the health of animals and our shared environment' (from: https://www.cdc.gov/onehealth/basics/index.html).

Do I have to participate?
No. Participation in this anonymous survey is voluntary and you can decide not to participate, not to answer all the questions or choose to end the survey at any time without giving a reason.

What would I be required to do?

If you accept this offer to participate, you will be invited to answer questions in an online survey.

The survey may take 20-30 minutes of your time, depending on how much detail you wish to provide.

What are the risks and benefits of participating in this research?
The anticipated risk of participating is the potential inconvenience of the survey taking up a short period of your time.

The anticipated benefits are improvements to the Regional One Health Network, which might translate to you, your region, your collaborations and/or your work.

Who will be able to access my information?
Your responses to this anonymous survey will be available to the research team: Dr Kirrilly Thompson, Dr Joanne Taylor, A/Prof Peter Massey and Prof David Durrheim from the Hunter New England Local Health District and the University of Newcastle.

If I take part, what happens to my information ?
Information will be collected via REDCap and stored in password protected Hunter New England Local Health District servers. You can view the REDCap privacy policy at: https://projectredcap.org/software/mobile-app/privacypolicy/

A de-identified summary of findings will be provided to participants and presented at a Regional One Health Network meeting for further discussion.

While you will not be named in research outcomes, it may be possible for others to identify you from your qualifications, organisation type, role and area of geographical impact.

We anticipate the preparation of a journal article will be submitted to a high-quality peer-review journal for the purpose of sharing key learnings to inform other One Health collaborative networks.

Who is conducting this research?
This research is being conducted by Dr Kirrilly Thompson, Dr Joanne Taylor, A/Prof Peter Massey and Prof David Durrheim from Hunter New England Local Health District and the University of Newcastle.

This research has no direct funding but is supported by Hunter New England Local Health District.

How to proceed
If you wish to proceed, please tick 'I agree' at the next question.

If you do not wish to proceed, please tick 'I do not agree' at the next question, or simply close this window.

More information is available from:
Dr Kirrilly Thompson, kirrilly.thompson@newcastle.edu.au or 0413 616 650.

Complaints Information
This research has been approved by the The University of Newcastle's Human Research Ethics Committee (HREC) Protocol Number H-2022-0098.

Should you have concerns about your rights as a participant in this research, or you have a complaint about the manner in which the research is conducted, it may be given to the researcher, or, if an independent person is preferred, please contact The Human Research

Ethics Officer on (02) 4921 6333 or via email at human-ethics@newcastle.edu.au

I agree to all of the following statements:

I am a current member of the Regional One Health Network, AND I have read the Information Statement about this research project, AND I am aware of the activities involved in the study, including any inconvenience that is currently known by the researchers, AND I can withdraw at any time without providing a reason, AND Due to the anonymous nature of the survey, any data submitted will not be able to be removed. I agree to participate in this research study.

◯ Yes ◯ No

Your experience working in/on One Health

How many years have you been working on One Health topics/issues? ______________________________

Please list the main One Health projects/programs/topics on which you are currently working _____________________________________

Please indicate which of the following concerns relate to any or all of the One Health projects/programs you listed in the preceding question

- ☐ Environment
- ☐ Human health
- ☐ Domestic animal/pet health
- ☐ Livestock health excluding horses
- ☐ Horses
- ☐ Wild animal health
- ☐ Acute disease
- ☐ Endemic disease
- ☐ One health systems
- ☐ One health surveillance
- ☐ Other

Please describe your "Other" concern here ______________________________

Please list any One Health projects/programs of work for which you are actively seeking collaboration and the kind of assistance required? (eg. disease, issue, methodology, region, field of work, species or 'none') _____________________________________

Your experience of the Regional One Health Network

How did you find out about the Regional One Health Network? __________________________________

What was/were your reason(s) for joining the Regional One Health Network? __________________________________

How many years have you been involved in the Regional One Health Network? ____________________________

Please list any other networks in which you participate that are relevant to One Health __________________________________

Benefits of the Regional One Health Network

What major One Health-related benefits have you gained from the Regional One Health Network in the past five years (list up to 3 or write 'none')? __________________________________

Has the Regional One Health Network provided you/your institution with exceptional One Health opportunities that would have otherwise not been possible?
- ◯ Yes
- ◯ No

Please describe those opportunities here ____________________________

Has being a part of the Regional One Health Network enabled you to develop any collaborations since you joined?
- ◯ Yes
- ◯ No

Can you please tell us how many Regional One Health Network collaborations and detail how they were initiated __________________________________

Please gauge the impact of your involvement in the R1HN on the following

	Not at all	Somewhat	Greatly	Unsure
The Regional One Health Network has assisted me/my institution with FORMING new One Health collaborations for research, surveillance or outbreak response	◯	◯	◯	◯
The Regional One Health Network has assisted me/my institution with STRENGTHENING existing One Health collaborations for research, surveillance or outbreak response	◯	◯	◯	◯
The Regional One Health Network has assisted me/my institution with being INFORMED of relevant One Health issues	◯	◯	◯	◯
The Regional One Health Network has contributed to POLICY CHANGE in my sector	◯	◯	◯	◯

Overall, how would you describe the usefulness of the Regional One Health Network to you?

◯ Not at all useful
◯ A little bit useful
◯ Fairly useful
◯ Very useful
◯ Unsure

The Regional One Health Network in the future

What are you hoping to achieve by participating in the Regional One Health Network (please select all that apply)?	☐ Improvement in One Health approach to the prevention and control of diseases relevant to humans, animals and the environment<br>☐ Research collaborations to address One Health issues<br>☐ One health training development and delivery<br>☐ Establishing connections between sectors to achieve One Health goals<br>☐ Other
Please tell us what other things you would like the Regional One Health Network to help you achieve.	__________________________________
Thinking of what you want to achieve with your One Health activities, how (else) do you think the Regional One Health Network can assist you?	__________________________________
How do you think the Regional One Health Network could be improved?	__________________________________
If the Regional One Health Network created a shared space for promoting activities and sharing information, which of the following do you think you would use (tick all that apply)?	☐ Facebook group<br>☐ LinkedIn group<br>☐ Sharepoint<br>☐ Google Drive<br>☐ Website<br>☐ Other
What other platform or shared space would you recommend?	__________________________________
What is your preferred form of communication with the Regional One Health Network?	◯ Email<br>◯ Online meetings<br>◯ fFace-to-face meetings<br>◯ Newsletters<br>◯ Social media<br>◯ Other
What is your other form of preferred communication for the Regional One Health Network?	__________________________________

Finally, we would like to collect some basic demographic and professional information

What is your gender?
- ◯ Female
- ◯ Male
- ◯ Non-binary
- ◯ Prefer not to say

Please list all of your qualifications relevant to One Health (please select all that apply)
- ☐ PhD
- ☐ Masters
- ☐ Honours
- ☐ Undergraduate/Bachelor degree
- ☐ Diploma
- ☐ Certificate
- ☐ Other [write in full below]

Please describe your "other" qualification

____

What are your areas of expertise? (Please be as specific as possible. Examples include: large animals, small animals, horses, dogs and cats, wildlife, human public health, surveillance, epidemiology, policy, etc.)

____

How is your organisation classified ?
- ☐ Local government or council
- ☐ State/Territory government
- ☐ Federal government
- ☐ Higher education
- ☐ Private industry
- ☐ Personal business/consultant
- ☐ NFP/Advocacy group
- ☐ Other (please state below)

Please describe the "other" classification here

____

What is your position in your organisation?

____

How would you classify your role in your organisation (please select all that apply)?
- ☐ Clinical
- ☐ Research
- ☐ Program development
- ☐ Education and/or training
- ☐ Prevention
- ☐ Fostering collaborations
- ☐ Policy
- ☐ Other (state at next question please)

Please describe your "other" role here

____

Which of these sectors/areas apply to your role?
- ☐ Human health
- ☐ Animal health
- ☐ Wildlife health
- ☐ Ecology
- ☐ Environmental health
- ☐ Cross cutting across sectors
- ☐ Other (please state below)

Please describe the "other" sector here

____

What geographic region(s)/local area(s) does your current work relate to? (eg. Scone, Central Australia, Alpine regions, NSW).

____

If you have any other comments about the Regional One Health Network, please use this box to let us know!

____
